# Strength Training and Insulin Resistance: The Mediating Role of Body Composition

**DOI:** 10.1155/2020/7694825

**Published:** 2020-05-08

**Authors:** McKayla J. Niemann, Larry A. Tucker, Bruce W. Bailey, Lance E. Davidson

**Affiliations:** Department of Exercise Sciences, Brigham Young University, Provo, Utah 84602, USA

## Abstract

The main objective of the present study was to assess the association between participation in strength training and insulin resistance. Another goal was to assess the influence of several potential confounding variables on the strength training and insulin resistance relationship. Lastly, the influence of waist circumference, fat-free mass (kg), body fat percentage, and the fat-free mass index on the association between strength training and insulin resistance was assessed. This cross-sectional study included 6,561 randomly selected men and women in the U.S. Data were collected using the precise protocol established by NHANES. HOMA-IR was used as the outcome variable to index insulin resistance. Both time spent strength training and frequency of strength training bouts were used as exposure variables. There was not a statistically significant relationship between strength training and insulin resistance in women. However, before and after controlling for 11 potential confounding variables, men who reported no strength training had significantly higher levels of HOMA-IR compared to men who reported moderate or high levels of strength training (*F* = 9.87, *P* < 0.0001). Odds ratios were also assessed. Men reporting no strength training had 2.42 times the odds of having insulin resistance compared to men reporting moderate levels of strength training (95% CI: 1.19-4.93). Similarly, men reporting no strength training had 2.50 times the odds of having insulin resistance compared to men reporting high levels of strength training (95% CI: 1.25-5.00). In conclusion, there was a strong relationship between strength training and insulin resistance in U.S. men, but not in U.S. women. Differences in waist circumference, fat-free mass (kg), body fat percentage, and the fat-free mass index, as well as demographic and lifestyle measures, do not appear to mediate the relationship. The present study was not a clinical trial.

## 1. Introduction

The Centers for Disease Control and Prevention estimates that 30.3 million people in the U.S. have diabetes, over 9% of the population [1]. Another 84.1 million adults are prediabetic, 33.9% of the adult population. Currently, diabetes is the 7^th^ leading cause of death in the United States [1]. Type 2 diabetes is also a major risk factor for coronary heart disease, the leading cause of mortality in the U.S [1]. It is also a significant predictor of stroke [1–3], atrial fibrillation [3, 4], and lower-extremity amputation [1].

Insulin resistance is a precursor to diabetes. People who are insulin resistant need greater amounts of insulin to get glucose into their cells [5]. If not corrected through physical activity, weight loss, and/or prescribed medication, insulin resistance frequently leads to type 2 diabetes.

Ample evidence suggests that physical activity can help prevent and treat insulin resistance and type 2 diabetes [6]. Physical activity lowers glucose levels in the blood by activating GLUT-4 vesicles, which transport glucose into the cells [7, 8]. In a recent review of studies conducted using a variety of designs, Bird and Hawley summarize numerous investigations, based on different physical activity durations and intensity levels and their effects on insulin sensitivity and glycemic control. The paper discusses molecular mechanisms for exercise-based changes in glucose uptake and insulin resistance. The authors conclude that acute improvements in insulin sensitivity occur after a single bout of exercise and chronic adaptations occur after at least 8 weeks of training [8].

Several investigations indicate that aerobic activities, such as jogging, walking, and cycling, reduce the risk of type 2 diabetes [9, 10]. However, far less research has been conducted on the relationship between strength training and insulin resistance. Strength training could be a favorable exercise option, especially for people with comorbid diseases, such as obesity or cardiac disease, which can make aerobic exercise challenging.

To date, a few investigations have shown an inverse, linear relationship between strength training and insulin resistance [11–13]. Some of the studies indicate that the relationship between strength training and insulin resistance is due to the body mass effect, which suggests that the muscle hypertrophy gained from strength training increases insulin sensitivity [13, 14]. However, other studies examining the mass effect have concluded that there is not a significant relationship between the increase in muscle mass due to strength training and insulin resistance [15, 16]. Additionally, some research indicates that differences in waist circumference have a meaningful influence on the strength training and insulin sensitivity relationship [17], whereas other research does not support the mediating role of abdominal adiposity [18].

Due to inconsistent findings and limited research, additional investigations are needed to assess the relationship between strength training and insulin resistance. Investigations designed to study the mediating effect of body composition, particularly fat-free mass and waist circumference, on the relationship between strength training and insulin resistance are especially needed.

The primary objective of the present study was to determine the extent to which varying amounts of strength training account for differences in insulin resistance in a nationally representative sample of U.S. adults. Another purpose was to determine the contribution of several potential confounding variables, including age, gender, race, year of assessment, smoking, body mass index, and participation in physical activities other than strength training, on the strength training and insulin resistance association. Lastly, and of particular interest, was the extent to which the relationship between strength training and insulin resistance was influenced by differences in body composition, particularly fat-free mass (kg), waist circumference, body fat percentage, and the fat-free mass index.

## 2. Methods

### 2.1. Design

A cross-sectional study using NHANES data was conducted. NHANES, the National Health and Nutrition and Examination Survey, is an extensive ongoing survey of health and nutrition information conducted by the National Center for Health Statistics. The National Center for Health Statistics is a part of the U.S. Centers for Disease Control and Prevention. Due to the use of fasting blood glucose and fasting insulin values to calculate HOMA-IR in the present study, and because NHANES has measured these key variables using different methods over the years, the present study focused on 8 years of NHANES data, from 1999 to 2006. Additional details pertaining to the methods used in the collection of NHANES data are available online [19].

Each survey participant submitted a written informed consent form to NHANES prior to data collection [20]. The Ethics Review Board for the National Center for Health Statistic (previously referred to as The Institutional Review Board) approved the NHANES data collection [20]. The Ethics Review Board also allowed the data files to be posted on the NHANES website for public use [20].

### 2.2. Subjects

A total of 6,561 participants were included in the present study. NHANES data were collected using a multi-stage random sample of noninstitutionalized people in the United States. The present investigation included participants aged 20-84 years. Although data were collected by NHANES on individuals older than 84 years, all subjects 85 years and older were given the age of 85 by NHANES to maximize confidentiality. Hence, these participants were not included in the current investigation. Each of the participants included had data on age, gender, race, year of assessment, HOMA-IR, minutes and sessions of strength training performed per week, MET-minutes of physical activity other than strength training, smoking status, BMI (body mass index), waist circumference, body fat percentage, and fat-free mass.

### 2.3. Instrumentation and Measurement Methods

#### 2.3.1. Race

NHANES classified race into 5 categories: Non-Hispanic White, Non-Hispanic Black, Mexican American, Other Race, and Other Hispanic.

#### 2.3.2. HOMA-IR

In the present investigation, insulin resistance was the outcome variable. HOMA-IR was used to index insulin resistance. The formula to calculate HOMA-IR was (fasting insulin (*μ*U/mL) × fasting glucose (mg/dL)/405). HOMA-IR is a common method of assessing insulin resistance. A literature search shows that over 22,000 studies use the term HOMA or HOMA-IR in their publications.

People with diabetes were not included in the study. Additionally, adults who were unable to give a blood sample, including those who had chemotherapy within 4 weeks, are hemophiliacs, and people with rashes, burns, edema, or paralysis, were not included [21].

Fasting insulin and fasting glucose information was obtained from NHANES data sets. Participants were asked to fast for nine hours prior to coming in for blood testing. To maximize the validity of the glucose and insulin results, before the blood sample, participants filled out a fasting survey that asked specific questions addressing the last time they had consumed food or liquids. Included in the questionnaire were questions specific to the ingestion of less commonly thought of foods such as breathe mints, gum, tea, alcohol, or supplements to ensure that the participants were following the fasting protocol. Blood samples of 89-92 mL were collected.

#### 2.3.3. Strength Training

The present study had three exposure variables, total minutes of strength training per month based on rounded hourly cut-points, total minutes of strength training per month based on sex-specific tertiles, and sessions of strength training per month. NHANES reported sessions of strength training per month, which was equivalent to days of strength training per month for a vast majority of the participants. A value of 4.3 weeks per month was used to convert monthly values to weekly for ease of interpretation (365 ÷ 12 = 30.4; 30.4 ÷ 7 = 4.3). Minutes of strength training per week were treated as a categorical variable. Adults who reported 10 minutes or less of strength training per week were put into a nonstrength training group. Adults who engaged regularly for more than 10 minutes of strength training per week were divided into 3 hour-based categories, according to the amount of time they trained per week—Low: 11-59 minutes per week, Moderate: 60-119 minutes per week, and High: 120 minutes or more per week. For men, there were 102 subjects in the Low category (4.1%), 81 in the Moderate group (3.0%), and 140 in the High category (5.3%). For women, there were 91 in the Low category (3.9%), 53 in the Moderate group (2.0%), and 36 women in the High category (1.6%).

A second exposure variable was also used based on sex-specific tertiles associated with minutes of strength training per week. Again, adults reporting 10 minutes or less of strength training per week were considered nonstrength trainers. Those reporting more than 10 minutes per week were then divided into tertiles. For men, 87.7% (*n* = 2,809) were considered nonstrength trainers, and 4.1% (*n* = 102) fit the Low tertile category, which included men reporting 11-59 minutes of strength training per week (equal to the categories based on simple hourly cut-points). A total of 4.1% of men (*n* = 110) were Moderate strength trainers, reporting 60-147 minutes of strength training per week, and 4.1% (*n* = 111) were in the Highest tertile category, which included men reporting more than 147 minutes of strength training per week. Given that NHANES assigned each participant an individual sample weight to allow maximum generalizability of the results, the percentages associated with each category more accurately represent the contribution of each subsample (e.g., 4.1%) than the subject count (e.g., *n* = 111).

For women, 92.5% (*n* = 3,249) were considered nonstrength trainers, and 2.4% (*n* = 58) fit the Low tertile category, which included women reporting 11-39 minutes of strength training per week. A total of 2.5% of women (*n* = 60) were Moderate strength trainers, reporting 40-81 minutes of strength training per week, and 2.6% (*n* = 62) were in the Highest tertile category, which included women reporting more than 81 minutes of strength training per week. Again, NHANES assigned each participant an individual sample weight to allow maximum generalizability of the results, so the percentages associated with each category more accurately represent the contribution of each subsample (e.g., 2.5%) than the subject count (e.g., *n* = 60).

A third exposure variable, sessions or bouts per week of strength training, was also treated as a categorical variable. Adults were divided into four groups: 0-1 session per week, 2 sessions per week, 3 sessions per week, and 4 or more sessions per week. For men, the distribution was 90.7% (*n* = 2,880), 2.1% (*n* = 53), 4.4% (*n* = 120), and 2.8% (*n* = 79), respectively. For women, the percentage and number in each category was 94.4% (*n* = 3,289), 2.1% (*n* = 48), 2.3% (*n* = 58), and 1.2% (*n* = 34), respectively.

#### 2.3.4. Other Physical Activity

Many people who participate in strength training also participate in other forms of physical activity (PA), which is why the present study used PA other than strength training as a covariate. NHANES collected frequency, duration, and intensity data on 47 physical activities other than strength training. Each physical activity was converted to MET minutes and then added together to index total MET minutes of other physical activity.

MET units are useful for describing energy expenditure of a specific activity and for comparing energy expenditure across multiple activities. A MET represents the ratio between the metabolic rate during PA and the metabolic rate at rest. To calculate MET minutes, the MET value of an activity was determined and was multiplied by the time spent engaged in the activity.

#### 2.3.5. Smoking

Smoking was indexed using pack-years. Pack-years are frequently used as an index of the amount an adult has smoked over several years. It is calculated by multiplying the number of years the person has smoked by the number of packs of cigarettes smoked per day. That number is then divided by 20, which is the number of cigarettes in each pack [22]. Smokers are more likely to develop insulin resistance than nonsmokers, which is why pack-years was used as a covariate in the present investigation [23].

#### 2.3.6. Height and Weight

Height was measured with each adults' heels, buttocks, shoulder blades, and back of the head against a wall [24]. The feet were flat on the floor with the toes angled outwards. A stadiometer was then used to measure standing height.

Participants were weighed on a digital Toledo scale [24]. When being weighed, participants were only wearing underwear, a disposable paper gown, and foam slippers [24]. Weight was recorded in pounds and then converted into kilograms. Both height and weight were used in the study to calculate body mass index (BMI).

#### 2.3.7. Body Mass Index

Body mass index (BMI) is a common measure of body weight independent of height. It is calculated as weight in kilograms divided by height in meters squared. It is frequently used as a measure of obesity. BMI is a good predictor of insulin resistance. Hence, BMI was used as a covariate in the present study. BMIs less than 18.5 are considered underweight. Normal weight BMIs are more than 18.5 and less than 25, whereas overweight BMIs are between 25 and less than 30. BMIs signifying obesity are 30 or more [25].

#### 2.3.8. Waist Circumference

Waist circumference was measured by finding the superior lateral iliac crest of the pelvis. The NHANES examiner then took the measurement at the iliac crest, holding the measuring tape parallel to the floor [24]. The measurement was taken to the nearest.1 cm after one normal exhalation of the participant. A steel measuring tape was put against the skin tightly, but without folding the skin. Waist circumference was used as a covariate in the present investigation because it is strongly correlated with insulin resistance [26].

#### 2.3.9. Body Fat Percentage

Body fat percentage was measured using DXA, dual-energy X-ray absorptiometry. DXA scans measure total body fat, lean body mass, and bone density. Women who were pregnant were not scanned. Also, due to the size of the scanner and room limitations, people who were over 6'5” or over 300 pounds were also not scanned. NHANES used multiple imputations to fill in the missing data. More information regarding how NHANES used multiple imputations can be found elsewhere [22]. The body fat percentage was used to calculate fat-free mass.

#### 2.3.10. Fat-Free Mass

Fat-free mass represents the amount of mass a person has that is not adipose tissue. Two measures of fat-free mass were employed in the present study: kilograms (kg) of fat-free mass and the fat-free mass index. The first was calculated by multiplying the body fat percentage by total mass (kg) and then subtracting this value (i.e., kg of fat mass) from total mass. The fat-free mass index was calculated by dividing kg of fat-free mass by height in meters squared. The fat-free mass index is much like BMI, but the outcome focuses only on body mass that is not fat, whereas BMI includes all mass. In most adults, fat-free mass is primarily muscle, although it also includes bone and connective tissues. Typically, the more adults participate in strength training, the more fat-free mass they have. Fat-free mass was used in this study because several investigations suggest that fat-free mass might play a critical role in the relationship between strength training and insulin resistance [12–14, 27].

### 2.4. Data Analysis

The NHANES sample that was used to generate the results of the present study was unique because participants were randomly selected from the adult population of the U.S. Therefore, findings can be generalized to the noninstitutionalized, civilian population because each participant was assigned a person-level sample weight. The sample weights, along with randomly selected strata and clusters, were included as part of each statistical analysis.

Given the large sample that was used in the present investigation, it is commonly assumed that the statistical power associated with each analysis is very high. However, this is not the case. Because of the multilevel sampling strategy used to acquire participants, the total number of degrees of freedom in the denominator for each analysis was 59, derived by subtracting the 28 strata from the 57 clusters. In short, because of nesting, statistical power associated with the present study was only moderate, at best.

Continuous variables were reported in Results as mean ± standard errors (SE). Outcomes for categorical variables were given as frequencies expressed as percentages ± SE. The descriptive statistics were determined using SurveyMeans and SurveyFreq, respectively.

HOMA-IR was the outcome variable for this cross-sectional investigation. There were three main exposure variables: (1) total minutes of strength training per month based on rounded hourly values; (2) total minutes of strength training based on sex-specific tertiles, for those reporting more than 10 minutes of strength training per week; and (3) sessions or bouts of strength training per month. Minutes per month and sessions per month were converted to minutes per week and sessions per week at times for easier interpretation. Minutes per week of strength training was treated as a categorical variable with four levels. Sessions per week of strength training were also treated as a categorical variable. Participants were assigned to one of four categories: 0-1 session per week, 2 sessions per week, 3 sessions per week, and 4 or more sessions per week of strength training.

The SurveyReg procedure was employed to determine the extent to which mean HOMA-IR levels differed across the strength training categories using multiple regression. To measure the extent to which mean HOMA-IR levels across the strength training categories were influenced by potential confounding factors including age, sex, race, year of assessment, smoking, BMI, other physical activity, body fat percentage, fat-free mass (kg), waist circumference, and the fat-free mass index, partial correlation was used. The covariates were controlled sequentially, first evaluating the effect of the demographic factors considered together, then the lifestyle variables were added to the model, and finally, the body composition variables were included in the model individually and finally together. To create adjusted means, the least square means procedure was used.

In another set of analyses, odds ratios were generated using SurveyLogistic to determine the odds of being insulin resistant (HOMA-IR ≥ 3:0) based on minutes of strength training per week. Effects of the demographic, lifestyle, and body composition covariates were also assessed.

For statistical significance, alpha was set at <0.05 and all *P* values were two-sided. To perform the statistical analyses, SAS version 9.4 was utilized (SAS Institute, Inc., Cary, NC, USA).

## 3. Results

NHANES sample weights were used in this study so that the findings are generalizable to men and women in the United States. In the present sample of 6,561 participants, the average HOMA-IR ± SE was 1.6 ± 0.03. Among adults who reported more than 10 minutes of strength training per week, the average number of strength training sessions per month was 12.0 ± 0.8 (2.8 ± 0.2 per week), and the mean number of minutes spent strength training per month was 284.7 ± 26.1 (66.2 ± 6.1 per week).  Table 1 further details characteristics of the sample of U.S. men and women used in this study.

In men, minutes (mean ± SE) of strength training per week were 1 (±0.9), 34 (±1.8), 83 (±2.7), and 247 (±11.4) for those in the nonstrength training, Low, Moderate, and High categories, respectively, after controlling for age, race, and year of assessment. For men based on tertiles of strength training, minutes (mean ± SE) of strength training per week were 1 (±0.8), 34 (±1.7), 96 (±3.5), and 277 (±10.7), respectively, after adjusting for the demographic variables. For women, the mean values were 0 (±0.2), 32 (±1.4), 82 (±2.5), and 177 (±9.8), respectively, after controlling for age, race, and year of assessment. For women based on tertiles of strength training, minutes (mean ± SE) of strength training per week were 0 (±0.3), 23 (±1.1), 55 (±1.7), and 145 (±7.9), respectively, after adjusting for the demographic variables.

Table 2 displays the mean differences in HOMA-IR and the amount of strength training participation between men and women. On average, mean HOMA-IR (±SE) was greater among men than women when including all subjects (*F* = 35.58, *P* < 0.0001) and when including only those who reported regular strength training (*F* = 7.4, *P* = 0.0088). The average number of strength training sessions per month (*F* = 26.24, *P* < 0.0001) and the mean minutes spent strength training per month (*F* = 51.19, *P* < 0.0001) were both higher in men than women. With the sample delimited to strength trainers, the average number of minutes spent strength training per month was also higher among men than women (*F* = 34.69, *P* < 0.0001). Other differences, not reported in Table 2, with all subjects included, were men participated in physical activities other than strength training more than women (*F* = 36.66, *P* < 0.0001). Men had lower levels of body fat than women (*F* = 4836.24, *P* < 0.0001). Men had larger waist circumferences than women (*F* = 251.94, *P* < 0.0001) and more fat-free mass (kg) than women (*F* = 6475.01, *P* < 0.0001). Lastly, men had a larger fat-free mass index than women (*F* = 3190.84, *P* < 0.0001).

Sessions per week of strength training were significantly related to HOMA-IR in men, but not in women. Specifically, men who reported strength training one or fewer sessions per week had significantly higher mean HOMA-IR levels than men who trained two, three, or four or more sessions per week. Mean differences were significant when the demographic variables were controlled (*F* = 12.0, *P* < 0.0001), and after adjusting for differences in the demographic covariates and the lifestyle covariates together (*F* = 11.3, *P* < 0.0001). With the body composition factors added to the other covariates, the relationship was weakened, but differences remained significant between men who trained one session or less per week compared to the others (*F* = 8.0, *P* = 0.0002). HOMA-IR means did not differ among men who reported strength training two, three, or four or more sessions per week.

Overall, the relationship between HOMA-IR and the level of strength training was only significant in men, which is why Tables 3 and 4 focus on men only.  Table 3 shows the odds of being insulin resistant (HOMA-IR ≥ 3:0; 75th percentile) in men who reported 10 minutes of strength training or less per week compared to men who reported low, moderate, or high levels of strength training per week, based on simple hourly cut-points.

As shown in Table 3, after controlling for the demographic covariates (age, race, and year of assessment), men who reported no strength training had 2.04 times the odds of being insulin resistant than men who reported a moderate level of strength training (95% CI: 1.02-4.08). Men who reported no strength training had 2.18 times the odds of being insulin resistant than men who reported a high level of strength training (95% CI: 1.24-3.86). Controlling for the demographic variables plus pack-years of smoking, other physical activity, and BMI increased the none vs moderate and none vs high odds ratios. Adding waist circumference to the covariates and then sequentially replacing waist circumference with fat-free mass (kg), body fat percentage, or the fat-free mass index had little effect on the odds ratios. Lastly, controlling for all the covariates together also had little effect on the odds ratios. Additionally, categorizing men according to their involvement in strength training based on tertiles, rather than simple hourly cut-points, had little effect on the odds ratios and did not change the statistical significance of any of the comparisons. In short, all the significant odds remained significant and all the nonsignificant odds ratios remained nonsignificant.

Table 4 shows the mean differences in HOMA-IR across categories of strength training in men based on hourly cut-points. After adjusting for the demographic covariates (age, race, and year of assessment), mean HOMA-IR values differed significantly between the no strength training category and both the moderate and high strength training categories (*F* = 11.40, *P* < 0.0001). Mean differences were similar with level of strength training based on tertiles, rather than hourly cut-points (*F* = 12.70, *P* < 0.0001). After controlling for the demographic variables plus the lifestyle covariates (i.e., pack-years of smoking, other physical activity, and BMI), the relationship between HOMA-IR and strength training remained significant between the no strength training group and both the moderate and high strength training categories (*F* = 13.88, *P* < 0.0001). Again, mean differences were similar with the level of strength training based on tertiles, rather than hourly cut-points (*F* = 15.34, *P* < 0.0001).

After adding waist circumference to the demographic and lifestyle covariates, the relationship remained significant (*F* = 12.65, *P* < 0.0001). Replacing the covariate, waist circumference, with fat-free mass (kg) (*F* = 11.98, *P* < 0.0001), body fat percentage (*F* = 11.91, *P* < 0.0001) or the fat-free mass index (*F* = 10.22, *P* < 0.0001) also resulted in significant associations. Lastly, all the covariates were controlled together and the relationship between strength training and HOMA-IR remained significant (*F* = 9.26, *P* < 0.0001).

Table 4 shows mean differences in HOMA-IR across categories of strength training based on simple hourly cut-points. Mean differences in HOMA-IR across levels of strength training based on tertiles are not shown in Table 4. When the analyses were conducted using categories based on tertiles, instead of hourly cut-points, each of the specific HOMA-IR comparisons across the strength training levels remained the same, significant, or not significant. Specifically, after adding waist circumference to the model as a covariate (*F* = 14.29, *P* < 0.0001), after substituting fat-free mass (kg) for waist (*F* = 13.33, *P* < 0.0001), after replacing waist with body fat percentage (*F* = 13.64, *P* < 0.0001), and after substituting the fat-free mass index for waist size (*F* = 11.92, *P* < 0.0001), each specific HOMA-IR comparison remained significant or nonsignificant as before. Likewise, basing the strength training categories on tertiles instead of hourly cut-points, and adjusting for differences in all the covariates together, each of the specific comparisons remained significant as before or nonsignificant as before (*F* = 10.63, *P* < 0.0001).

## 4. Discussion

The purpose of this study was three-fold: first, to assess the extent to which varying levels of strength training account for differences in insulin resistance within a random sample of 6,561 noninstitutionalized adults in the United States; second, to determine the influence of several mediating factors on the association between strength training and insulin resistance; and third, to evaluate the extent that multiple measures of body composition influence the strength training and insulin resistance association.

Mean differences between men and women were evaluated. On average, men had higher HOMA-IR, more strength training sessions per month, and more minutes spent strength training per month than women. Moreover, men had more fat-free mass (kg), a larger mean waist size, and larger average fat-free mass index, and men reported engaging in more physical activity other than strength training, than women. Women had a higher body fat percentage than men. Due to the significant differences between women and men, the relationship between strength training and insulin resistance was studied in men and women separately.

There was not a significant relationship between strength training and insulin resistance in women. In contrast, results showed robust significance in men. The gender differences may be due to the varying levels of strength training between men and women. The Centers for Disease Control and Prevention reported that in the U.S., 21.5% of men and 17% of women participate in two or more days of strength training [28]. Moreover, and of greater importance, according to the present study's national data, men who strength trained reported about 6 hours and 20 minutes of resistive exercise per month, whereas women who strength trained reported about 4 hours per month. Similarly, the 75^th^ percentile for minutes of strength training per month was almost 13 hours for men, whereas for women, it was about 6 hours and 45 minutes, a substantial difference. Men participating in higher levels of strength training than women may be why the association between strength training and HOMA-IR was stronger in men than in women in the present study. There do not appear to be gender differences in physiological responses to strength training [29–31], at least when considered after months of training.

According to a literature review by Bird and Hawley, when an exercise program fails to improve insulin sensitivity, as found in the subsample of women in the present study, it may be that the exercise intensity was too low, the duration was too short, or the group already had good insulin sensitivity, and therefore, the potential to improve was limited [8]. All three of these issues could explain why time spent strength training in U.S. women was not related to insulin sensitivity. Although intensity was not measured, men reported more than a 50% higher strength training duration than women. Moreover, average level of HOMA-IR was more than 30% higher in men strength trainers compared to women strength trainers. Accordingly, strength training may not have benefitted U.S. women to the same degree as U.S. men because women already had much better insulin sensitivity.

After controlling for the demographic covariates (age, race, and year of assessment), the results showed that men who reported 10 minutes or less of strength training per week had about 2 and a half times the odds of having insulin resistance compared to those with moderate or high levels of strength training. The results also indicated that the mean HOMA-IR values were significantly higher in the no strength training group compared to both the moderate and high strength training groups. However, men reporting low levels of strength training (<1 hour per week) did not differ from those reporting no strength training. Because of the cross-sectional design of the present study, causation cannot be concluded. However, 2 and a half times greater odds suggests a strong relationship.

Controlling for the lifestyle covariates (i.e., pack-years of smoking, other physical activity, and BMI), along with the demographic covariates, increased the none vs moderate and none vs high odds ratios. Also, the mean HOMA-IR values remained significantly different between the no strength training group and both the moderate and high strength training groups.

There are multiple mechanisms that could account for the association between strength training and insulin resistance in men. The present investigation controlled for waist circumference, fat-free mass (kg), body fat percentage, and the fat-free mass index to assess the potential mediating role of each variable. Controlling for the demographic and lifestyle covariates and adding waist circumference as a potential mediating factor had little effect on the association. The mean HOMA-IR values remained significantly higher in the no strength training group compared to both the moderate and high strength training groups.

Other investigations have also found that waist circumference has no effect on the relationship between strength training and insulin resistance. Grøntved et al. conducted a cross sectional study of 32,002 men from the Health Professionals Follow-Up Study [17]. Weight training resulted in a 48% reduced risk of developing type two diabetes [17]. Waist circumference weakened the correlation between strength training and type 2 diabetes, but the association remained statistically significant [17]. Dunstan et al. also considered the role of waist circumference in an eight-week circuit training protocol on 27 individuals [32]. It was concluded that circuit training decreased glucose levels independent of changes in waist circumference [32].

A few studies have concluded that waist circumference plays a mediating role in the association between exercise and insulin resistance. However, most studies have assessed aerobic exercise, not strength training. For example, in a cross-sectional study, Garciá-Hermoso et al. concluded that waist circumference fully mediates the relationship between physical activity and HOMA-IR [33]. Lee et al., who assessed resistance training along with aerobic training in a randomized controlled trial with 40 teenaged boys, found that changes in abdominal fat were significantly related to both resistance training and aerobic training and increased insulin sensitivity [34].

Adjusting for differences in the demographic and lifestyle covariates and adding fat-free mass (expressed as kg or the fat-free mass index) as a potential mediator of the strength training and HOMA-IR association had little effect. Several studies support the present investigation's finding. A quasiexperimental study including 28 overweight males who participated in a 12-week resistance training program found that, on average, the men increased in lean body mass [16]. However, lean body mass did not appear to mediate the relationship between strength training and insulin resistance [16]. Additionally, Anderson et al. assessed insulin resistance after 90 days of training followed by 90 days of detraining [35]. Changes in glucose clearance were expressed relative to muscle mass, measured using magnetic resonance imaging. Results showed that detraining increased insulin resistance and decreased fat-free mass [35]. Anderson et al. concluded that strength training was associated with decreases in insulin resistance and that fat-free mass did not play a role in the association [35]. Lastly, Holten et al. established a single-leg six-week training program among 10 men with diabetes and seven healthy age-matched controls [15]. Results showed that glucose clearance increased in the trained leg compared to the untrained leg in both the men with diabetes and the healthy controls [15]. Muscle mass and fiber size did not increase significantly; therefore, it was concluded that strength training increases glucose clearance in the absence of changes in muscle mass [15].

There are a few studies suggesting that fat-free mass does mediate the relationship between strength training and insulin resistance. In a six-month trial assessing both aerobic and strength training, Poehlman et al. concluded that strength training increases insulin sensitivity by increasing fat-free mass [14]. In a 16-week strength training study, results showed a decrease in insulin resistance and an increase in muscle fiber size, or hypertrophy, suggesting that the decrease in insulin resistance after 16 weeks of strength training was due to muscle hypertrophy [13]. Hypertrophy may increase the amount of skeletal muscle tissue and GLUT 4 receptors [13, 14]. Given the mixed findings in the literature, additional research is needed to determine the mediating role of lean body mass or fat-free mass in the relationship between strength training and insulin resistance.

Controlling for the demographic and lifestyle covariates and adding body fat percentage to the model had little effect on the results. These findings are consistent with the literature. Poehlman et al. assessed body fat percentage and insulin sensitivity in a six-month exercise protocol and found no change in body fat percentage [14]. It was concluded that body fat percentage does not mediate the relationship between strength training and insulin sensitivity. Moreover, in an eight-week trial, Dunstan et al. did not see any changes in body fat percentage and reached a similar conclusion as Poehlman et al. that body fat percentage does not play a role in the strength training and insulin resistance association [32].

Theoretically, there are multiple mechanisms that could account for the association between strength training and insulin resistance, including waist circumference, fat-free mass, and body fat percentage. However, findings of the present investigation did not identify any of these factors as significant mediators. Other potential mechanisms, not measured in this study, could play a role. For example, the association could be due to increases in signaling proteins involved in glucose clearance because of strength training. Holten et al. applied a single-leg resistance training intervention among 10 men and concluded that strength training decreased insulin resistance beyond the effects of fat-free mass [15]. Holten et al. measured several proteins and saw an increase in GLUT 4, protein kinase B, and glycogen synthase [15]. Ahtiainen et al. also used leg resistance training among men and found that resistance training increases signaling proteins [36]. Specifically, Ahtiainen et al. measured several proteins via muscle biopsy 30 minutes preexercise and 30 minutes postexercise and concluded that IRS-1 signaling is downgraded while the AMPK pathway is upregulated, which activates A160 [36].

After controlling for all the covariates simultaneously, age, race, year of assessment, pack years of smoking, other physical activity, BMI, waist circumference, fat-free mass (kg), body fat percentage, and the fat-free mass index, the none vs moderate and none vs high levels of strength training odds ratios changed minimally. In short, men reporting moderate to high levels of strength training remained much less likely to be insulin resistant compared to their counterparts. Furthermore, the mean HOMA-IR values remained significantly higher and more unfavorable in the no strength training group compared to both the moderate and high strength training groups. Apparently, if U.S. men all had the same age, race, year of assessment, pack years of smoking, other physical activity, BMI, waist circumference, fat-free mass (kg), body fat percentage, and fat-free mass index, those reporting no regular strength training would tend to have more insulin resistance compared to their counterparts.

This study had some limitations. Strength training was self-reported, so there could be misclassification of strength training amounts, which could influence the findings. However, self-reporting is likely to weaken the association rather than strengthen it. Additionally, participants who reported high levels of strength training could have other lifestyle factors that are unique to them. Lastly, due to the cross-sectional design of this study, results cannot be interpreted as causal.

The study also had several strengths, including a large sample size of 6,561. Eleven potential confounding factors were assessed and controlled. Lastly, the results are generalizable to all noninstitutionalized men and women in the U.S due to the random selection of participants by NHANES.

## 5. Conclusion

In conclusion, levels of insulin resistance differed significantly between U.S. men reporting no strength training and those reporting moderate or high levels of strength training. There was not a significant difference between U.S. men reporting no strength training and those reporting low levels of strength training. Moreover, there was not a significant relationship between strength training and insulin resistance in U.S. women. Even after adjusting for differences in numerous potential confounding factors, men reporting no strength training had about 2 and a half times the odds of being insulin resistance compared to men reporting either moderate or high levels of strength training. Finally, in the present study, the association between strength training and insulin resistance in U.S. men was not influenced by differences in waist circumference, fat-free mass (kg), body fat percentage, or the fat-free mass index.

## Figures and Tables

**Table 1:** Percentiles for the key variables representing U.S. women and men.

Variable	Percentile (±SE)				
	5th	25th	50th	75^th^	95^th^
HOMA-IR					
Women (*n* = 3429)	0.3 ± 0.02	0.9 ± 0.02	1.5 ± 0.04	2.5 ± 0.07	5.6 ± 0.2
Men (*n* = 3132)	0.4 ± 0.02	1.0 ± 0.02	1.7 ± 0.03	3.0 ± 0.08	6.4 ± 0.3
Combined (*n* = 6561)	0.4 ± 0.01	0.9 ± 0.02	1.6 ± 0.03	2.8 ± 0.05	6.0 ± 0.2
Waist circumference (cm)					
Women (*n* = 3429)	71.1 ± 0.3	80.7 ± 0.3	90.0 ± 0.5	102.1 ± 0.5	120.4 ± 1.1
Men (*n* = 3132)	76.6 ± 0.7	89.1 ± 0.4	97.5 ± 0.3	107.3 ± 0.5	124.5 ± 1.1
Combined (*n* = 6561)	72.8 ± 0.4	84.3 ± 0.3	94.2 ± 0.3	105.0 ± 0.4	122.4 ± 0.7
Fat-free mass (kg)					
Women (*n* = 3429)	32.5 ± 0.2	37.8 ± 0.2	42.0 ± 0.2	47.1 ± 0.2	56.5 ± 0.5
Men (*n* = 3132)	47.0 ± 0.4	55.3 ± 0.3	61.2 ± 0.3	67.5 ± 0.3	79.1 ± 0.6
Combined (*n* = 6561)	34.4 ± 0.2	41.6 ± 0.2	50.8 ± 0.3	61.2 ± 0.3	74.3 ± 0.4
Body fat %					
Women (*n* = 3429)	28.7 ± 0.4	35.9 ± 0.2	41.2 ± 0.2	45.7 ± 0.2	51.3 ± 0.3
Men (*n* = 3132)	16.6 ± 0.3	23.7 ± 0.2	28.0 ± 0.2	32.2 ± 0.2	38.5 ± 0.3
Combined (*n* = 6561)	19.0 ± 4.3	27.6 ± 0.2	34.2 ± 0.4	41.8 ± 0.2	49.3 ± 0.3
BMI (kg/m^2^)					
Women (*n* = 3429)	19.4 ± 0.2	22.9 ± 0.1	26.5 ± 0.2	31.5 ± 0.25	40.6 ± 0.5
Men (*n* = 3132)	20.4 ± 0.1	24.2 ± 0.1	27.1 ± 0.1	30.4 ± 0.1	37.4 ± 0.5
Combined (*n* = 6561)	19.8 ± 0.1	23.5 ± 0.1	26.8 ± 0.1	31.0 ± 0.1	39.5 ± 0.4
Fat-free mass index (kg/m^2^)					
Women (*n* = 3429)	13.0 ± 0.1	14.4 ± 0.1	15.8 ± 0.1	17.5 ± 0.1	20.9 ± 0.2
Men (*n* = 3132)	16.0 ± 0.1	18.0 ± 0.1	19.6 ± 0.1	21.2 ± 0.1	24.2 ± 0.2
Combined (*n* = 6561)	13.5 ± 0.0	15.5 ± 0.1	17.7 ± 0.1	19.9 ± 0.1	23.2 ± 0.1
Other PA (min)					
Women (*n* = 3429)	0.0 ± 15.7	0.0 ± 15.7	250.6 ± 29.8	1097.3 ± 65.2	3515.7 ± 160
Men (*n* = 3132)	0.0 ± 15.5	0.0 ± 15.5	439.9 ± 39.0	1466.8 ± 55.6	5123.6 ± 244
Combined (*n* = 6561)	0.0 ± 13.6	0.0 ± 13.6	328.2 ± 30.7	1259.9 ± 47.2	4392.8 ± 216
ST sessions/month					
Women (*n* = 3429)	0.0 ± 0.9	0.0 ± 0.9	0.0 ± 0.9	0.0 ± 0.9	8.2 ± 1.3
Men (*n* = 3132)	0.0 ± 0.8	0.0 ± 0.8	0.0 ± 0.8	0.0 ± 0.8	12.5 ± 1.0
Combined (*n* = 6561)	0.0 ± 0.7	0.0 ± 0.7	0.0 ± 0.7	0.0 ± 0.7	12.0 ± 0.9
ST min/month (min)					
Women (*n* = 3429)	0.0 ± 5.0	0.0 ± 5.0	0.0 ± 5.0	0.0 ± 5.0	171.1 ± 30.2
Men (*n* = 3132)	0.0 ± 5.1	0.0 ± 5.1	0.0 ± 5.1	0.0 ± 5.1	527.9 ± 91.9
Combined (*n* = 6561)	0.0 ± 4.8	0.0 ± 4.8	0.0 ± 4.8	0.0 ± 4.8	273.1 ± 35.2
ST sessions/month^∗^					
Women (*n* = 180)	2.1 ± 0.2	6.0 ± 1.0	8.9 ± 0.9	12.9 ± 0.9	21.0 ± 1.7
Men (*n* = 323)	1.5 ± 0.9	7.1 ± 0.9	12.2 ± 0.8	14.2 ± 0.8	26.7 ± 1.7
Combined (*n* = 503)	1.8 ± 0.1	7.0 ± 0.8	12.0 ± 0.8	13.0 ± 0.8	25.9 ± 1.6
ST min/month^∗^ (min)					
Women (*n* = 180)	56.4 ± 0.0	129.5 ± 12.3	239.8 ± 20.2	404.4 ± 48.4	823.8 ± 55.2
Men (*n* = 323)	56.2 ± 0.0	187.2 ± 25.1	378.9 ± 33.4	776.0 ± 56.6	1555.5 ± 57
Combined (*n* = 503)	57.1 ± 1.8	150.1 ± 13.4	284.7 ± 26.1	603.5 ± 58.3	1523.4 ± 90

SE: standard error. Table values include person-level weighted adjustments based on the sampling methods of NHANES so that values represent those of the U.S. adult population. ^∗^The sample was delimited to individuals reporting more than 10 minutes of strength training per week. Men: *n* = 323, women: *n* = 180.

**Table 2:** Mean differences in HOMA and amount of strength training between U.S. men and women after adjusting for covariates (*n* = 6561).

	Men (*n* = 3132)	Women (*n* = 3429)	*F*	*P*
	Mean ± SE	Mean ± SE		
HOMA-IR	2.61 ± 0.09	2.20 ± 0.07	35.6	<0.0001
ST sessions per month	1.24 ± 0.12	0.59 ± 0.11	26.2	<0.0001
ST min. per month	64.95 ± 8.62	17.62 ± 5.29	51.2	<0.0001
ST sessions per month^∗^	13.08 ± 0.56	12.32 ± 0.74	1.1	0.2900
ST min. per month^∗^	638.58 ± 61.42	416.50 ± 51.55	34.7	<0.0001
HOMA-IR^∗^	2.01 ± 0.12	1.54 ± 0.17	7.4	0.0088

^∗^The sample was delimited to individuals reporting more than 10 minutes of strength training per week. Men: *n* = 323, women: *n* = 180. SE: standard error; HOMA-IR: homeostatic model assessment of insulin resistance. ST sessions per month: number of strength training sessions reported each month. ST min. per month: minutes of strength training reported each month. Means have been adjusted for the covariates: age, race, and year of assessment. The *F* and *P* values are based on 59 degrees of freedom.

**Table 3:** Odds of insulin resistance in men reporting no strength training compared to higher amounts of strength training based on hourly cut-points.

Outcome: HOMA (75^th^ percentile) Variable controlled:	None vs Low		None vs Moderate		None vs High	
	OR	95% CI	OR	95% CI	OR	95% CI
Model 1	1.69	0.95-3.00	2.04	1.02-4.08	2.18	1.24-3.86
Model 2	1.62	0.84-3.11	2.69	1.33-5.46	2.95	1.48-5.89
Model 3	1.57	0.82-3.00	2.55	1.26-5.13	2.64	1.33-5.28
Model 4	1.59	0.84-3.05	2.65	1.30-5.40	2.90	1.45-5.83
Model 5	1.55	0.81-2.95	2.45	1.19-5.04	2.60	1.31-5.16
Model 6	1.54	0.81-2.94	2.48	1.21-5.10	2.64	1.33-5.25
Model 7	1.58	0.84-3.00	2.47	1.21-5.01	2.60	1.30-5.20

OR = odds ratio; odds of having insulin resistance (HOMA ≥ 75^th^ percentile). 95% CI = 95% confidence interval. For the categories representing minutes of strength training, None included men reporting ≤10 minutes per week of strength training. Low included men reporting >10 minutes per week and <60 minutes per week, Moderate included men reporting ≥60 minutes per week and <120 minutes per week, and High included men reporting ≥120 minutes per week. Odds ratios on the same line as a model were adjusted for potential covariates in that model. Model 1 included age, race, and year of assessment as covariates. Model 2 included age, race, year, pack-years of smoking, other physical activity, and BMI. Model 3 included the same covariates as Model 2 plus waist circumference. Model 4 included the same covariates as Model 2 plus fat-free mass (kg). Model 5 included the same covariates as Model 2 plus body fat percentage. Model 6 included the same covariates as Model 2 plus the fat-free mass index. Model 7 included the same covariates as Model 2 plus waist circumference, fat-free mass (kg), body fat percentage, and the fat-free mass index.

**Table 4:** Mean differences in HOMA-IR in men across categories of strength training based on simple, hourly cut-points, after adjusting for potential confounders.

Outcome variable	Minutes of strength training								*F*	*P*
	None		Low		Moderate		High			
	Mean	SE	Mean	SE	Mean	SE	Mean	SE		
HOMA-IR										
Model 1	2.59^a^	0.12	2.24^a,b^	0.36	1.96^b^	0.21	1.94^b^	0.20	11.40	<0.0001
Model 2	2.62^a^	0.08	2.37^a,b^	0.32	2.00^b^	0.17	1.94^b^	0.12	13.88	<0.0001
Model 3	2.66^a^	0.09	2.45^a,b^	0.32	2.07^b^	0.17	2.10^b^	0.11	12.65	<0.0001
Model 4	2.61^a^	0.07	2.37^a,b^	0.32	2.01^b^	0.16	1.95^b^	0.10	11.98	<0.0001
Model 5	2.62^a^	0.08	2.38^a,b^	0.32	2.03^b^	0.17	2.00^b^	0.12	11.91	<0.0001
Model 6	2.62^a^	0.07	2.42^a,b^	0.31	2.08^b^	0.15	2.09^b^	0.10	10.22	<0.0001
Model 7	2.66^a^	0.09	2.58^a,b^	0.32	2.15^b^	0.15	2.23^b^	0.09	9.26	<0.0001

^a,b^Means on the same row with the same superscript letter are not significantly different (*P* > 0.05). SE = standard error of the mean. Means have been adjusted according to the covariates included in the model. Model 1 included age, race, and year of assessment as covariates. Model 2 included age, race, year, pack-years of smoking, other physical activity, and BMI. Model 3 included the same covariates as Model 2 plus waist circumference. Model 4 included the same covariates as Model 2 plus fat-free mass. Model 5 included the same covariates as Model 2 plus body fat percent. Model 6 included the same covariates as Model 2 plus the fat-free mass index. Model 7 included the same covariates as Model 2 plus waist circumference, fat-free mass (kg), body fat percentage, and the fat-free mass index. For the categories representing minutes of strength training, None included men reporting ≤10 minutes per week of strength training. Low included men reporting >10 minutes per week and <60 minutes per week, Moderate included men reporting ≥ to 60 minutes per week and < 120 minutes per week, and High included men reporting ≥120 minutes per week.

## Data Availability

All data used in the present study are available online as part of the National Health and Nutrition Examination Survey (NHANES). The data are free and can be accessed by using the following Centers for Disease Control and Prevention website: https://wwwn.cdc.gov/nchs/nhanes/Default.aspx
